# Changes in functional connectivity among vestibulo-visuo-somatosensory and spatial cognitive cortical areas in persistent postural-perceptual dizziness: resting-state fMRI studies before and after visual stimulation

**DOI:** 10.3389/fneur.2023.1215004

**Published:** 2023-07-24

**Authors:** Chihiro Yagi, Yuka Morita, Tatsuya Yamagishi, Shinsuke Ohshima, Shuji Izumi, Kuniyuki Takahashi, Masaki Watanabe, Kosuke Itoh, Yuji Suzuki, Hironaka Igarashi, Arata Horii

**Affiliations:** ^1^Department of Otolaryngology Head and Neck Surgery, Niigata University Graduate School of Medical and Dental Sciences, Niigata, Japan; ^2^Department of Otolaryngology Head and Neck Surgery, Faculty of Medicine, University of Miyazaki, Miyazaki, Japan; ^3^Center for Integrated Human Brain Science, Brain Research Institute, Niigata University, Niigata, Japan

**Keywords:** persistent postural-perceptual dizziness, resting-state functional magnetic resonance imaging, functional connectivity, visual stimuli, vestibular system, chronic dizziness

## Abstract

**Introduction:**

Persistent postural-perceptual dizziness (PPPD) is a functional chronic vestibular syndrome with symptom exacerbation by upright posture, motion, and complex visual stimuli. Among these exacerbating factors, visual exacerbation is the most specific characteristic of PPPD requiring further investigation. We hypothesized that stimulus-induced changes occur in the functional connectivity (FC) rather than simple neural activation that is involved in visual stimulation. The present study aimed to identify the neural basis of PPPD by investigating FC before and after visual stimulation.

**Methods:**

Eleven patients with PPPD and 11 age- and sex-matched healthy controls (HCs) underwent resting-state fMRI (rs-fMRI) before and after task-based fMRI with visual stimuli.

**Results:**

At pre-stimulus, FC between the vestibular cortex and visual areas was low, while that between the somatosensory and visual areas was high in PPPD compared with that in HCs. FC between the visuospatial (parahippocampal gyrus) and spatial cognitive areas (inferior parietal lobule) was elevated in PPPD even in the pre-stimulus condition, which no longer increased at post-stimulus as observed in HCs. In the post-stimulus condition, FC between the visual and spatial cognitive areas and that between the visual and prefrontal areas increased compared with that in the pre-stimulus condition in PPPD. Task-based fMRI demonstrated that no brain regions showed different activities between the HC and PPPD groups during visual stimulation.

**Discussion:**

In PPPD, vestibular inputs may not be fully utilized in the vestibulo-visuo-somatosensory network. Given that the FC between visuospatial and spatial cognitive areas increased even in HCs after visual stimuli, elevated status of this FC in combination with the high FC between the somatosensory and visual areas would be involved in the visual exacerbation in PPPD. An increase in FC from the visual areas to spatial cognitive and prefrontal areas after visual stimuli may account for the prolonged symptoms after visual exacerbation and anxious status in PPPD.

## 1. Introduction

Persistent postural-perceptual dizziness (PPPD) is a functional vestibular disorder characterized by chronic vestibular symptoms lasting over 3 months. The core symptoms are dizziness, unsteadiness, and non-spinning vertigo that are exacerbated by three factors: upright posture or walking, active or passive movement, and exposure to moving or complex visual stimuli (1).

Persistent postural-perceptual dizziness is usually preceded by conditions that disrupt balance or cause acute or episodic vertigo, unsteadiness, or dizziness. The most common preceding conditions are peripheral or central vestibular disorders (1, 2). Posture is maintained by three sensory inputs: visual, vestibular, and somatosensory information. Preceding vestibular disorders disrupt balance and posture, leading to two reactions: first, heightened vigilance as expressed by postural stiffness during standing and walking, which is also observed in healthy individuals when standing on elevated or unstable surfaces (3–5), and second, increased reliance on visual and/or somatosensory information (6, 7). Generally, these two conditions return to normal with the recovery of the preceding disease. However, the psychological trend of patients with PPPD involving neuroticism or introversion (8, 9) could influence the persistence of these conditions (10). Sustained heightened vigilance and increased reliance on visual and/or somatosensory information cause persistent dizziness and exacerbation by visual stimuli and motions (11). Ultimately, these processes may alter the spatial orientation (12) and impair postural control in complex environments (13, 14).

Recent neuroimaging studies on PPPD have gradually revealed the neural mechanisms that account for the abovementioned pathophysiological models. Resting-state functional MRI (rs-fMRI) and voxel-based morphometry have shown reduced functional connectivity (FC) and decreased gray matter volume, respectively, in multimodal vestibular cortical areas of patients with PPPD compared with those of healthy controls (HCs) (15, 16). Among three exacerbating factors, visual exacerbation is the most specific characteristic of PPPD (17) and requires further investigation. Therefore, we focused on the neural mechanisms underlying visual exacerbation in this study. Once symptom exacerbation by visual stimuli occurs, it persists for hours or more, suggesting that the stimulus-induced changes occur in FC rather than the simple neural activation that is involved in the visual stimulation. Hence, we performed rs-fMRI on patients with PPPD and normal volunteers before and after visual stimulation. In addition, task-based fMRI analysis was also performed during visual stimulation.

## 2. Methods

### 2.1. Patients

Eleven patients with PPPD were enrolled in this study between October 2020 and September 2021. As a control group, 11 healthy volunteers who were matched for age, sex, and handedness to patients with PPPD were included. All healthy volunteers had no history of vertigo or dizziness and no serious medical diseases.

Persistent postural-perceptual dizziness was diagnosed using the diagnostic criteria of the Barany Society (1). The Japanese version of the dizziness handicap inventory (DHI) (18, 19) was used to assess the severity of vestibular symptoms, the Hospital Anxiety and Depression Scale (HADS) (20) to evaluate anxiety and depression levels, the Japanese version of the Ten Item Personality Inventory (TIPI-J) (21, 22) to assess personality, the Visual Analog Scale (VAS) to evaluate the degree and changes in vestibular symptoms before and after the visual stimuli, and the Simulator Sickness Questionnaire (SSQ) (23) to evaluate visual stimulation-induced symptoms.

To assess the patients’ vestibular function, bithermal caloric testing, rotatory chair test (RCT), video head impulse test (vHIT), cervical and ocular vestibular-evoked myogenic potential (cVEMP and oVEMP, respectively), and subjective visual vertical (SVV) test were conducted. Bithermal caloric testing was performed by stimulating each external auditory canal twice with air at 26°C and 45°C for 60 s at 5-min intervals. The maximum slow phase velocity was measured using an electronystagmography and canal paresis (CP; %) was calculated using Jongkee’s index formula (24). RCT was done with a rotatory chair to which a pendulum-like rotation was applied, so that the maximum head angular velocity was 50^°^/s at a stimulation frequency of 0.1 Hz. The eye movements were monitored using an electronystagmography and vestibulo-ocular reflex (VOR) gain was calculated. vHIT was conducted using EyeSeeCam^®^ (Interacoustics, Denmark) to assess the VOR gain and corrective catch-up saccades (CUS) during a rapid high-velocity head turn. cVEMP and oVEMP using the Neuropack System^®^ (Nihon Kohden, Japan) were performed to evaluate the otolithic function, and the interaural asymmetry ratios (IAARs) of the cVEMP and oVEMP were used as indicators of saccular and utricular function, respectively. SVV test was also conducted to assess otolithic function using the SVV examination system (UNIMEC, Japan). CP greater than 25%, VOR gain less than 0.3 in RCT (25), VOR gain less than 0.8 with CUS in vHIT (26), IAAR greater than 32% (27, 28), and SVV greater than 2.5 degrees (29) were considered abnormal values.

All participants underwent static posturography on a solid or foam rubber surface using Gravicoda^®^ (ANIMA Corp., Japan) with eyes open and in closed conditions. The elliptical balance area (cm^2^) was adopted as a representative index of the degree of postural sway. Pure tone audiometry, blood pressure measurement, and blood routine tests were performed if indicated.

### 2.2. Experimental design and visual stimuli

**Figure 1:**
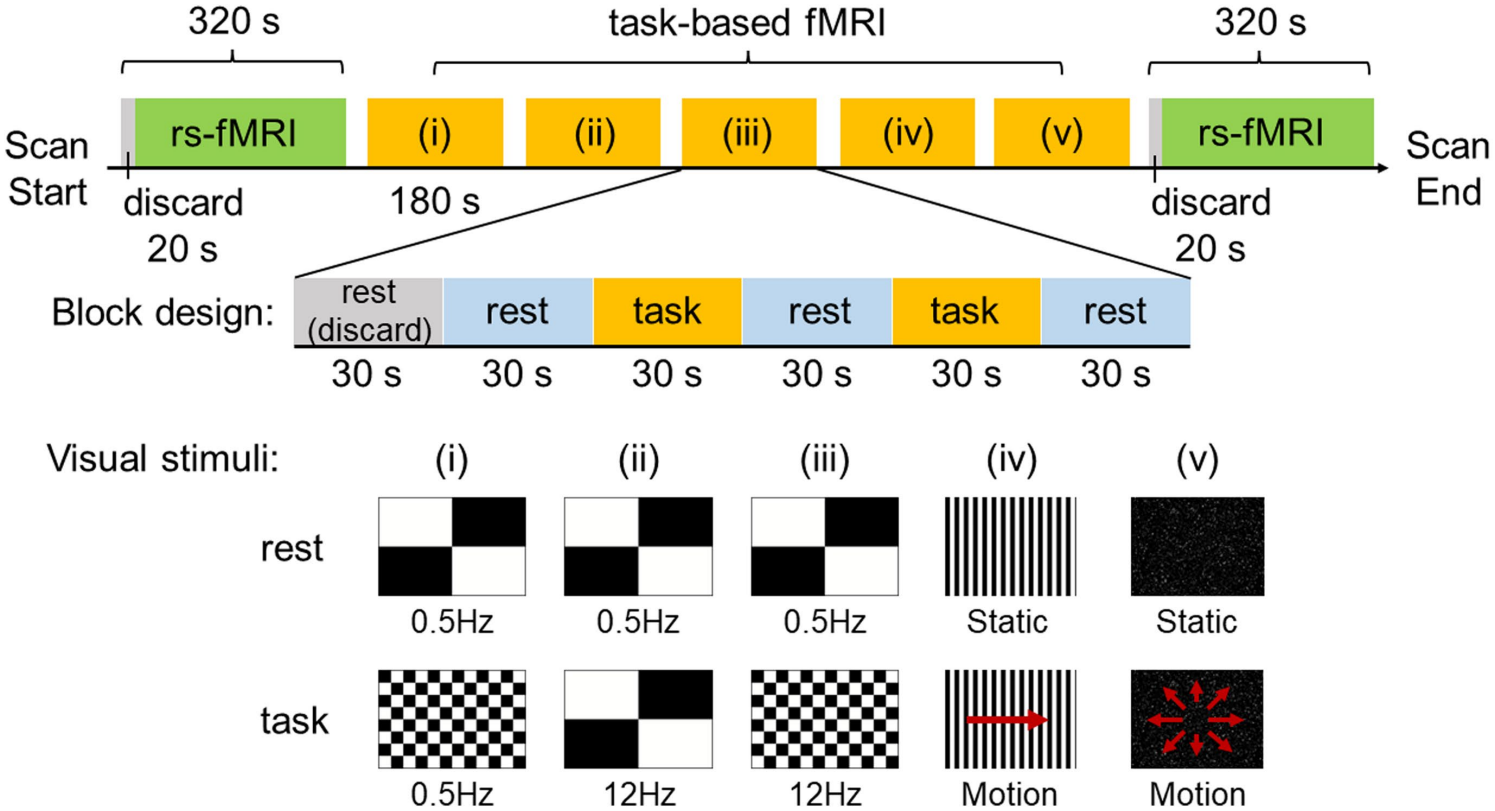
Experimental design and visual stimuli. Resting-state functional MRI (rs-fMRI) was acquired before and after visual stimulation for 320 s each and the data for the initial 20 s was discarded to ensure a steady state. The visual stimulation task used in this study utilized a block design and consisted of five different visual stimuli. One block consisted of 180 s, with the data for the initial 30 s discarded to ensure a steady state. Rest and task were administered alternately for 30 s each.

Rs-fMRI was acquired before (pre-stimulus) and after (post-stimulus) visual stimulation for 320 s each (Figure 1). The data from the initial 20 s were discarded to ensure a steady state. The visual stimulation task designed and used in this study consisted of five different visual stimuli. Each block consisted of 180 s, and the data for the initial 30 s were discarded to ensure a steady state. The rest and task were administered alternately for 30 s each.

The visual stimuli used in this study were created with the After Effects software (Adobe Inc., San Jose, CA, United States) by attempting to reproduce the stimuli of scenes that are likely to exacerbate symptoms in patients with PPPD in daily life, such as flashing lights on TV, scenery flowing sideways when viewed from inside a train, and scenery flowing from front to back when riding in a passenger car. In the experiments, visual stimuli consisted of (i) a checkerboard pattern stimulus comprising 8 rows × 12 columns of squares reversed in contrast (100%) at 0.5 Hz, (ii) a checkerboard pattern stimulus comprising 2 rows × 2 columns of squares reversed in contrast (100%) at 12 Hz, (iii) a checkerboard pattern stimulus comprising 8 rows × 12 columns of squares reversed in contrast (100%) at 12 Hz, (iv) optokinetic stimulus by 12 black-and-white vertical stripes sweeping across a screen at 6°/s, and (v) radial optic flow stimulus with moving white dots (size: 0.1–1.1 degrees of visual angle, speed: 3°/s with a flat speed gradient) on a black background expanding from the center of the screen. For visual stimuli (i), (ii), and (iii), a checkerboard pattern comprising 2 rows × 2 columns of squares reversed in contrast (100%) at 0.5 Hz was presented as rest. For visual stimuli (iv) and (v), the respective static images were presented as rest.

### 2.3. Imaging

All imaging data were acquired on the Signa LX 3.0-Tesla (GE Medical System) imaging system with an 8-channel head coil. During image acquisition, the participants were instructed to relax, stay awake, and focus on the middle of the screen throughout the experiment. As a general quality assurance procedure, functional scans were checked for head movements with a translation not exceeding 0.6 mm in any axis during each run. If a head movement exceeding 0.6 mm was observed, the run was re-performed. The structural images were recorded using a three-dimension spoiled gradient recalled echo (3D-FSPGR) sequence [repetition time (TR), 7.4 ms; field of view (FOV), 200 × 200 mm^2^; voxel size, 0.781 × 0.781 × 1.5 mm^3^; matrix, 256 × 256; echo time (TE), 3.04 ms; flip angle, 20^°^; slice thickness, 1.5 mm; slice spacing, 1.5 mm]. The functional images were obtained using gradient-echo echo-planar pulse sequence (TR, 1000 ms; FOV, 200 × 200 mm^2^; voxel size, 3.125 × 3.125 × 7.5 mm^3^; matrix, 64 × 64; TE, 30 ms; flip angle, 70^°^; slice thickness, 5 mm; slice spacing, 7.5 mm).

### 2.4. Preprocessing

#### 2.4.1. rs-fMRI

The rs-fMRI images were preprocessed using Statistical Parametric Mapping 12 (SPM12, Wellcome Department of Cognitive Neurology, United Kingdom) and the CONN toolbox (version 21a; http://www.nitrc.org/projects/conn) working on MATLAB R2022a (MathWorks, Inc., Natick, United States). The preprocessing and quality assurance of functional and structural MRI data were performed according to the default pipeline implemented in CONN as follows: (a) realignment and unwarp, (b) slice timing correction, (c) outlier detection with conservative settings (95th percentile of the normative sample), (d) segmentation and normalization (transform to the Montreal Neurological Institute [MNI] space), and (e) smoothing using a 6-mm fullwidth at half-maximum (FWHM) Gaussian kernel. After the preprocessing, time points were identified as outliers if movement from a preceding image exceeded a 0.5 mm deviation or global mean signal intensity exceeded 3 standard deviations. These time points were included as regressors along with principal components extracted from anatomical noise regions and realignment parameters during a denoising step. Finally, a band-pass filter was applied to the functional data with a frequency window of 0.008–0.09 Hz.

#### 2.4.2. Task-based fMRI

The task-based fMRI images were preprocessed using the SPM12 software. Functional images were realigned to the first image in the series to correct for within-scan head motions, performed slice timing correction to correct for temporal misalignment of slices, coregistered with the T1-weighted structural image for each subject, normalized to the MNI space, and spatially smoothed by an 8-mm FWHM Gaussian kernel.

### 2.5. Data analysis

#### 2.5.1. Demographic and clinical characteristics

To compare the demographic and clinical characteristics between the HC and PPPD groups, the Mann–Whitney *U* test was performed for HADS, TIPI-J, SSQ, and posturographic data. Statistical significance was set at *p* < 0.05. Statistical analyses were performed using GraphPad Prism version 9 (GraphPad Software, San Diego, CA, United States).

#### 2.5.2. rs-fMRI analysis

We performed seed-to-voxel resting-state FC analysis using priori-defined seed regions related to the vestibular, visual, somatosensory, and spatial cognitive regions of the brain. For the vestibular cortex, we selected the parieto-insular vestibular cortex (PIVC) and posterior insular cortex (PIC). The seed regions were determined as spheres with a radius of 5 mm, according to the latest structural study (30): *x* = −36, *y* = −25, *z* = 18 for the left PIVC; *x* = 36, *y* = −22, *z* = 17 for the right PIVC; *x* = −46, *y* = −33, *z* = 24 for the left PIC; and *x* = 51, *y* = −27, *z* = 28 for the right PIC. For the visual cortex, we selected the intracalcarine cortex (ICC), supracalcarine cortex (SCC), lingual gyrus (LG), and cuneal cortex (CC) bilaterally. For the somatosensory cortex, we selected the post-central gyrus (PostCG) bilaterally. For the visuospatial and spatial cognitive regions, we selected the anterior/posterior parahippocampal gyrus (aPaHC and pPaHC) and hippocampus (HC), respectively. The above seeds related to visual, somatosensory, and spatial cognition were determined from the atlas, which consists of cortical or subcortical regions of interest from the FSL Harvard–Oxford Atlas and is included by default in CONN.

To infer clusters of voxels functionally connected to each seed region, two thresholds were sequentially applied based on the random field theory method, used with a cluster-forming threshold of uncorrected *p* < 0.001 and cluster-level threshold of *p* < 0.05 corrected for multiple comparisons by using family-wise error (FWE).

To compare the base FC conditions between patients with PPPD and HCs, a two-sample *t*-test was performed on the pre-stimulus data.

For comparisons between pre- and post-stimulus in the PPPD group, data from only patients in whom dizziness symptoms were exacerbated by the visual stimuli, confirmed by an increase in the VAS score compared with that of pre-stimulus, were used. Differences in FC between pre- and post-stimulus in HCs or PPPD were tested using a paired *t*-test.

**Figure 2:**
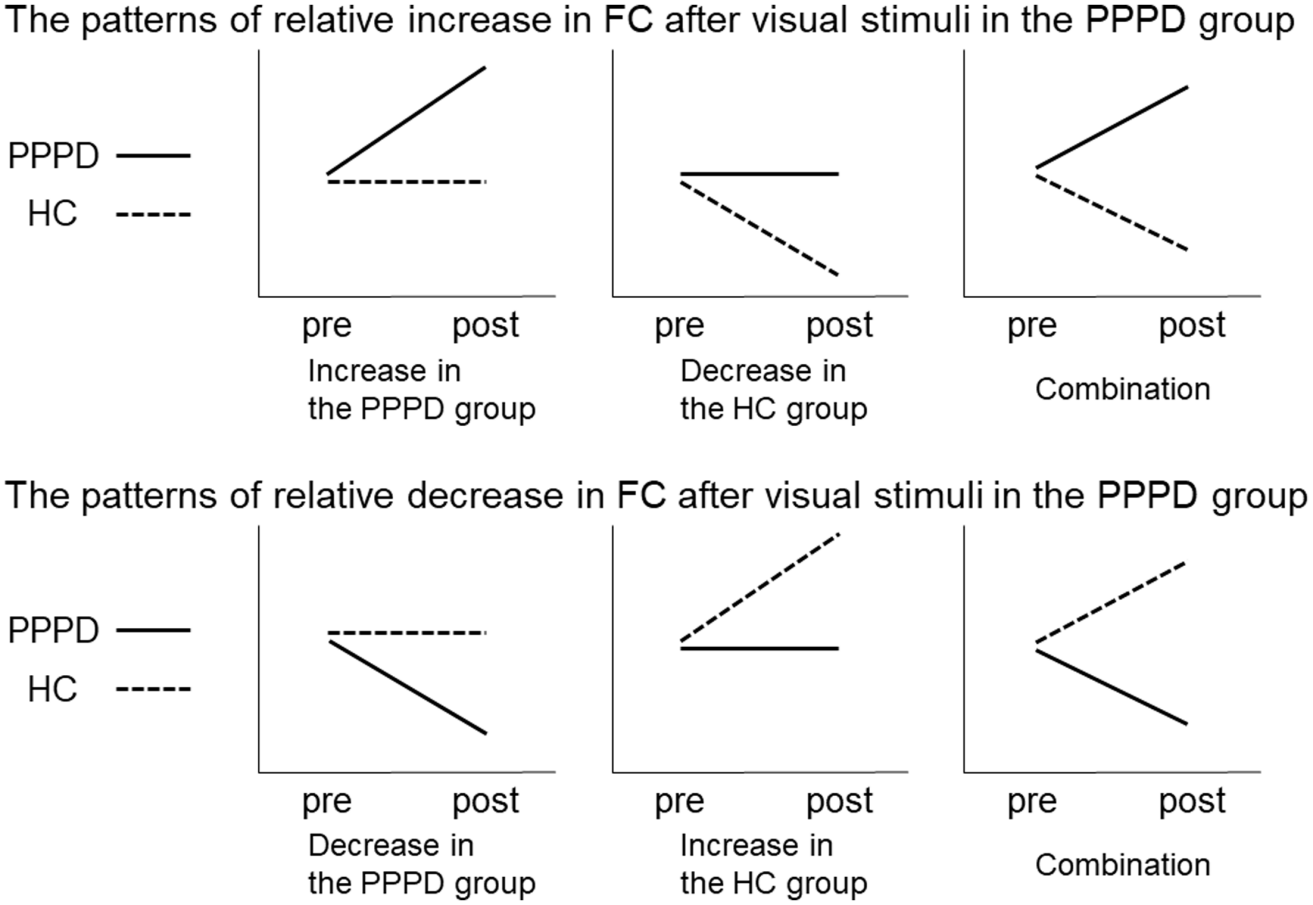
Relative increase or decrease in FC after visual stimuli in the PPPD group. Patterns of changes in PPPD/HC group showed significant differences between the pre- and post-stimulus conditions in the PPPD group relative to the HC group. The upper and lower row shows the patterns of relative increase or decrease after visual stimulation in the PPPD group, respectively. FC, functional connectivity; HC, hippocampus; PPPD, persistent postural-perceptual dizziness.

To detect regions showing a significant change in FC after visual stimuli in the PPPD group, differences between pre- and post-stimulus were evaluated relative to those of the HC group. This was tested using a 2 × 2 mixed ANOVA interaction. The patterns of changes in PPPD/HC group showed significant differences between the pre- and post-stimulus conditions in the PPPD group relative to the HC group (Figure 2). When the patterns of the relative increase in FC are observed after visual stimuli in the PPPD group, the following three possibilities may be included: increase in the PPPD group, decrease in the HC group, and a combination of both (the upper row). Similarly, when the patterns of the relative decrease in FC are observed after visual stimuli in the PPPD group, the following three possibilities may be included: decrease in the PPPD group, increase in the HC group, and a combination of both (the lower row).

For all group analyses, we used FWE-corrected values for multiple comparisons, and *p* < 0.05 was considered to indicate statistical significance.

#### 2.5.3. Task-based fMRI analysis

For the first-level analysis, the onsets and durations of the task were modeled, and the change in brain activity during the task relative to that during rest was set as a contrast. For the second-level analysis, the group analyses with unpaired *t*-tests comparing the HC and PPPD groups were performed with a significant threshold at FWE-corrected *p* < 0.05.

### 2.6. Ethics statement

This study was approved by the institutional review board of Niigata University Medical and Dental Hospital (Niigata city, Japan; #2019-0021). All procedures performed in this study were in accordance with the ethical standard of the 1964 Helsinki Declaration. Informed consent was obtained from all participants at the time of inclusion in the study, authorizing the anonymous use of data for further studies.

## 3. Results

### 3.1. Demographic and clinical characteristics

Table 1 summarizes the demographic and clinical data of the HC and PPPD groups. Two males and nine females were included in each group. All participants were right-handed. There was no significant difference in age between the two groups. As shown in Table 1, the Mann–Whitney *U* test demonstrated that the HADS score (total score) and the neuroticism score of the TIPI-J were significantly higher in the PPPD group than in the HC group. There was no significant difference in the elliptical balance area (with eyes open) between the two groups, while the elliptical balance area (with eyes closed, eyes open on foam rubber, eyes closed on foam rubber) of the PPPD group was significantly larger than that of the HC group. The SSQ score of the PPPD group was significantly higher than that of the HC group.

**Table 1:** Demographic profiles and characteristics of the healthy controls (HCs) and patients with persistent postural-perceptual dizziness (PPPD).

Variables	HCs	PPPD	*Value of p* (Mann–Whitney *U* test)
Sample size (male/female)	11 [2/9]	11 [2/9]	
Age, years	46 (8)	42 (4)	0.30
HADS (total score)	9 (7)	16 (14)	<0.01^**^
TIPI-J Extraversion	4.5 (3.0)	2.5 (3.5)	0.07
Agreeableness	5.0 (1.5)	4.5 (2.5)	0.39
Conscientiousness	4.0 (2.0)	4.0 (1.5)	0.66
Neuroticism	3.5 (2.0)	6.0 (2.5)	<0.01^**^
Openness	3.5 (3.0)	4.0 (2.5)	0.21
Elliptical balance area, cm^2^ Eyes open	3.3 (3.7)	6.1 (3.9)	0.19
Eyes closed	4.5 (3.8)	9.3 (7.9)	0.02^*^
Eyes open on foam rubber	6.1 (2.5)	9.6 (5.1)	<0.01^**^
Eyes closed on foam rubber	10.6 (5.6)	17.6 (15.6)	<0.01^**^
SSQ (total score)	3.7 (18.7)	44.9 (78.5)	<0.01^**^

*Values indicate statistical significance. ^*^p < 0.05; ^**^p < 0.01.

Values are reported as median and interquartile range.

HADS, Hospital Anxiety and Depression Scale; HCs, healthy controls; PPPD, persistent postural-perceptual dizziness; SSQ, Simulator Sickness Questionnaire; TIPI-J, Ten Item Personality Inventory.

Table 2 summarizes the demographic data of each patient with PPPD. The median duration of disease was 32 months (interquartile ranges [IQR]: 14 months), and the preceding diseases were acute unilateral vestibulopathy (AUVP) in 6 patients, benign paroxysmal positional vertigo (BPPV) in 4 patients, and chronic anxiety disorders in 1 patient. The median DHI score was 34 (IQR: 48). Of the 11 patients with PPPD, 5 were taking escitalopram or venlafaxine. Eight of 11 patients had exacerbation of dizziness symptoms by visual stimulation, which was confirmed by an increase in the VAS score compared with that of pre-stimulus data. Data from only these eight exacerbated patients were used for comparisons between pre- and post-stimulus in the PPPD group. In contrast, no participant in the HC group complained of dizziness symptoms during/after visual stimuli.

**Table 2:** Demographic characteristics of patients with persistent postural-perceptual dizziness (PPPD).

Patient no.	Age (years)	Sex (M/F)	Duration (months)	Preceding disease	DHI	Medication	VAS (before → after)/Exacerbation (+ or –)
P01	43	F	32	AUVP	10	Escitalopram	5 → 7/(+)
P02	41	F	27	BPPV	82	(−)	3 → 8/(+)
P03	42	F	7	AUVP	34	(−)	2 → 6/(+)
P04	42	F	33	AUVP	12	Venlafaxine	3 → 3/(−)
P05	45	M	34	BPPV	50	(−)	6 → 6/(−)
P06	39	F	5	BPPV	74	(−)	5 → 7/(+)
P07	41	F	50	AUVP	48	Escitalopram	0 → 2/(+)
P08	47	M	73	AUVP	26	(−)	4 → 7/(+)
P09	32	F	23	AUVP	64	Venlafaxine	7 → 9/(+)
P10	44	F	35	BPPV	30	Escitalopram	1 → 1/(−)
P11	40	F	6	Chronic anxiety disorders	16	(−)	4 → 5/(+)

AUVP, acute unilateral vestibulopathy; BPPV, benign paroxysmal positional vertigo; DHI, Dizziness Handicap Inventory; F, female; HADS, Hospital Anxiety and Depression Scale; M, male; PPPD, persistent postural-perceptual dizziness; VAS, visual analog scale.

Vestibular test results for patients with PPPD are shown in Supplementary Table 1. Some patients (P01, P06, and P09) showed results deviating from the normal range; however, no cases of obvious peripheral vestibular dysfunction were found based on the overall findings of the examination.

### 3.2. Pre-stimulus FCs in PPPD and HCs

**Figure 3:**
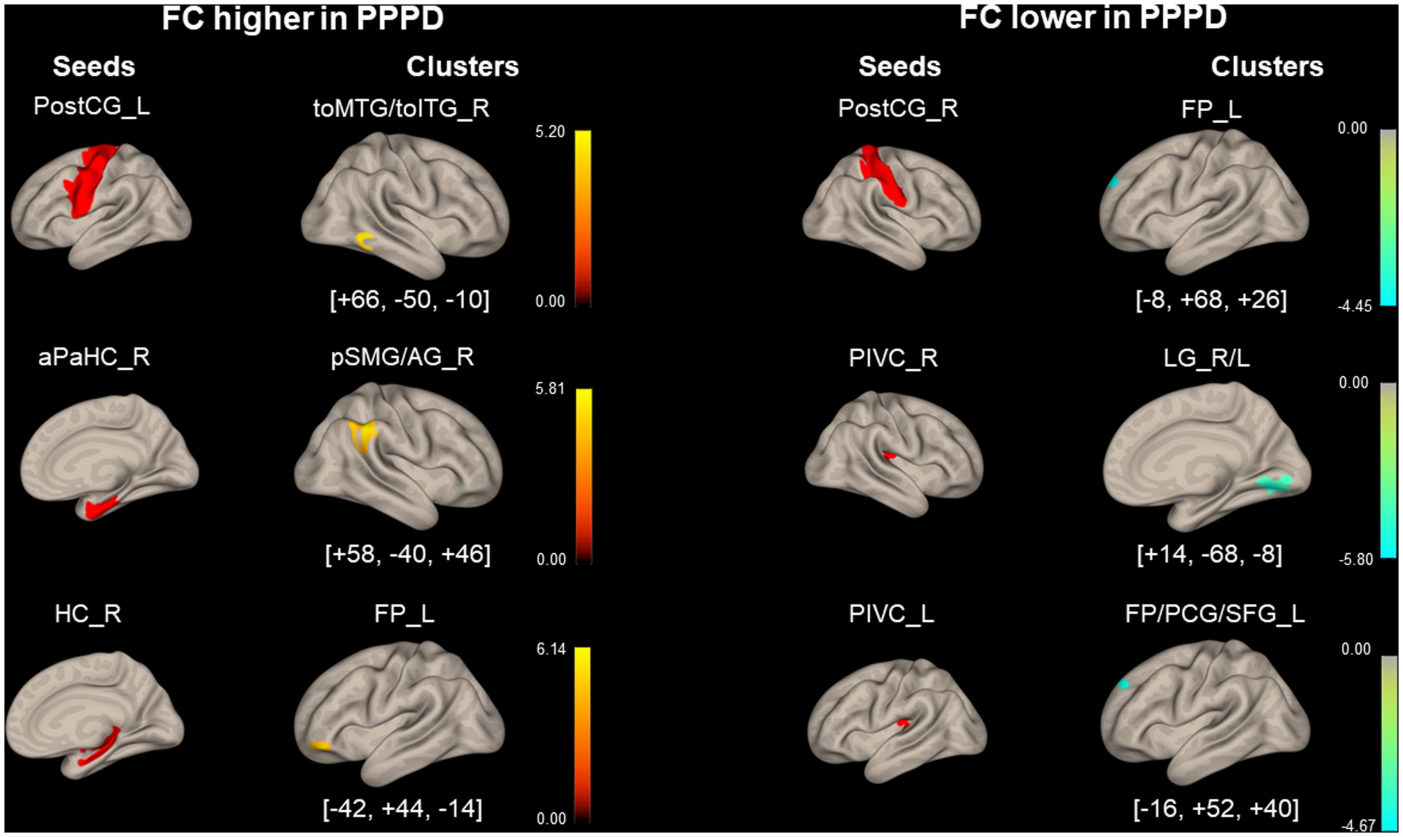
Significantly different functional connectivity between patients with persistent postural-perceptual dizziness and healthy controls under the pre-stimulus condition. Seed regions are shown in red, higher functional connectivity (FC) is indicated by yellow bars, and lower FC is indicated by green bars. The color bar represents *T* scores. The [*x*, *y*, *z*] values indicate the Montreal Neurological Institute (MNI) coordinates. aPaHC, anterior parahippocampal gyrus; FP, frontal pole; FP/PCG/SFG, frontal pole/paracingulate gyrus/superior frontal gyrus; HC, hippocampus; LG, lingual gyrus; PIVC, parieto-insular vestibular cortex; PPPD, persistent postural-perceptual dizziness; pMTG, posterior middle temporal gyrus; postCG, post-central gyrus; pSMG/AG, posterior supramarginal gyrus/angular gyrus; toMTG/toITG, temporooccipital middle/inferior temporal gyrus.

The PPPD group showed several significant differences in FC compared with the HC group at pre-stimulus. FC between the left PostCG and the right temporooccipital part of the middle/inferior temporal gyrus (toMTG/toITG), right aPaHC and the right posterior division of supramarginal gyrus/angular gyrus (pSMG/AG), and the right HC and the left frontal pole (FP) in the PPPD group was significantly higher than that in the HC group at pre-stimulus (Figure 3; Table 3). Conversely, FC between the right PostCG and the left FP, the right PIVC and the bilateral LG, and the left PIVC and the left FP/paracingulate gyrus/superior frontal gyrus (FP/PCG/SFG) in the PPPD group was significantly lower than those in the HC group (Figure 3; Table 3).

**Table 3:** Significantly different functional connectivity (FC) between patients with persistent postural-perceptual dizziness (PPPD) and healthy controls (HCs) in the pre-stimulus condition.

Seed region	Cluster coordinates (x, y, z)	Cluster size	Cluster regions	Cluster *value of p* (FWE)
*Higher FC in PPPD*				
Postcentral Gyrus_L	+ 66– 50– 10	138	Middle/Inferior Temporal Gyrus, temporooccipital part_R	0.015
Parahippocampal Gyrus, anterior division_R	+ 58– 40+ 46	589	Supramarginal Gyrus, posterior division_R/Angular Gyrus_R	<0.001
Hippocampus_R	− 42 + 44– 14	215	Frontal Pole_L	<0.001
*Lower FC in PPPD*				
Postcentral Gyrus_R	− 8 + 68 + 26	145	Frontal Pole_L	<0.01
PIVC_R	+ 14– 68– 8	738	Lingual Gyrus_R/L	<0.001
PIVC_L	− 16 + 52 + 40	140	Frontal Pole_L/Paracingulate Gyrus_L/Superior Frontal Gyrus_L	<0.01

R/L, bilateral; FC, functional connectivity; FWE, family-wise error; HCs, healthy control; L, left; PIVC, parieto-insular vestibular cortex; PPPD, persistent postural-perceptual dizziness; R, right.

### 3.3. Differences between pre- and post-stimulus FCs

**Figure 4:**
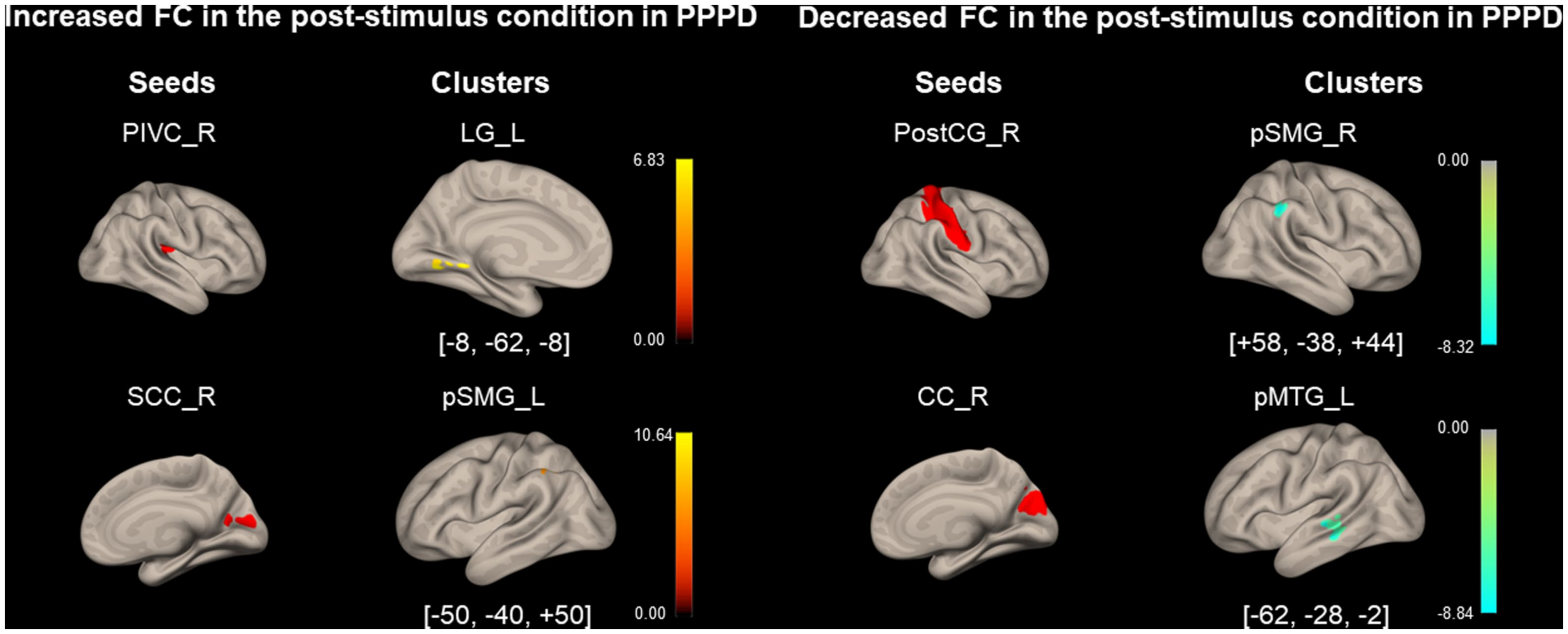
Significantly modified functional connectivity under the post-stimulus condition in the persistent postural-perceptual dizziness group. Seed regions are shown in red, increased functional connectivity (FC) is indicated by yellow bars, and decreased FC is indicated by green bars. The color bar represents *T* scores. The [*x*, *y*, *z*] values indicate the Montreal Neurological Institute (MNI) coordinates. CC, calcarine cortex; LG, lingual gyrus; PIVC, parieto-insular vestibular cortex; PPPD, persistent postural-perceptual dizziness; pMTG, posterior middle temporal gyrus; postCG, post-central gyrus; pSMG, posterior supramarginal gyrus; SCC, supracalcarine cortex.

As shown in Figure 4 and Table 4, FC between the right PIVC and left LG and that between the right SCC and left pSMG significantly increased in the post-stimulus condition than those in the pre-stimulus condition in the PPPD group. FC between the right PostCG and right pSMG and that between the right CC and left pMTG significantly decreased in the post-stimulus condition than those in the pre-stimulus condition in the PPPD group. No FC other than these 4 FCs showed significant changes after the stimulus compared with those before the stimulus in PPPD.

**Table 4:** Significantly modified functional connectivity (FC) in the post-stimulus condition in the persistent postural-perceptual dizziness (PPPD) group.

Seed region	Cluster coordinates (x, y, z)	Cluster size	Cluster regions	Cluster *value of p* (FWE)
*Increased FC*				
PIVC_R	−8 -62 -8	98	Lingual Gyrus_L	<0.001
Supracalcarine Cortex_R	−50 -40 + 50	77	Supramarginal Gyrus, posterior division_L	<0.01
*Decreased FC*				
Postcentral Gyrus_R	+58–38 + 44	99	Supramarginal Gyrus, posterior division_R	<0.01
Cuneal Cortex _R	−62 -28 -2	119	Posterior Middle Temporal Gyrus_L	<0.001

FC, functional connectivity; PPPD, persistent postural-perceptual dizziness; FWE, family-wise error; L, left; PIVC, parieto-insular vestibular cortex, R, right.

**Figure 5:**
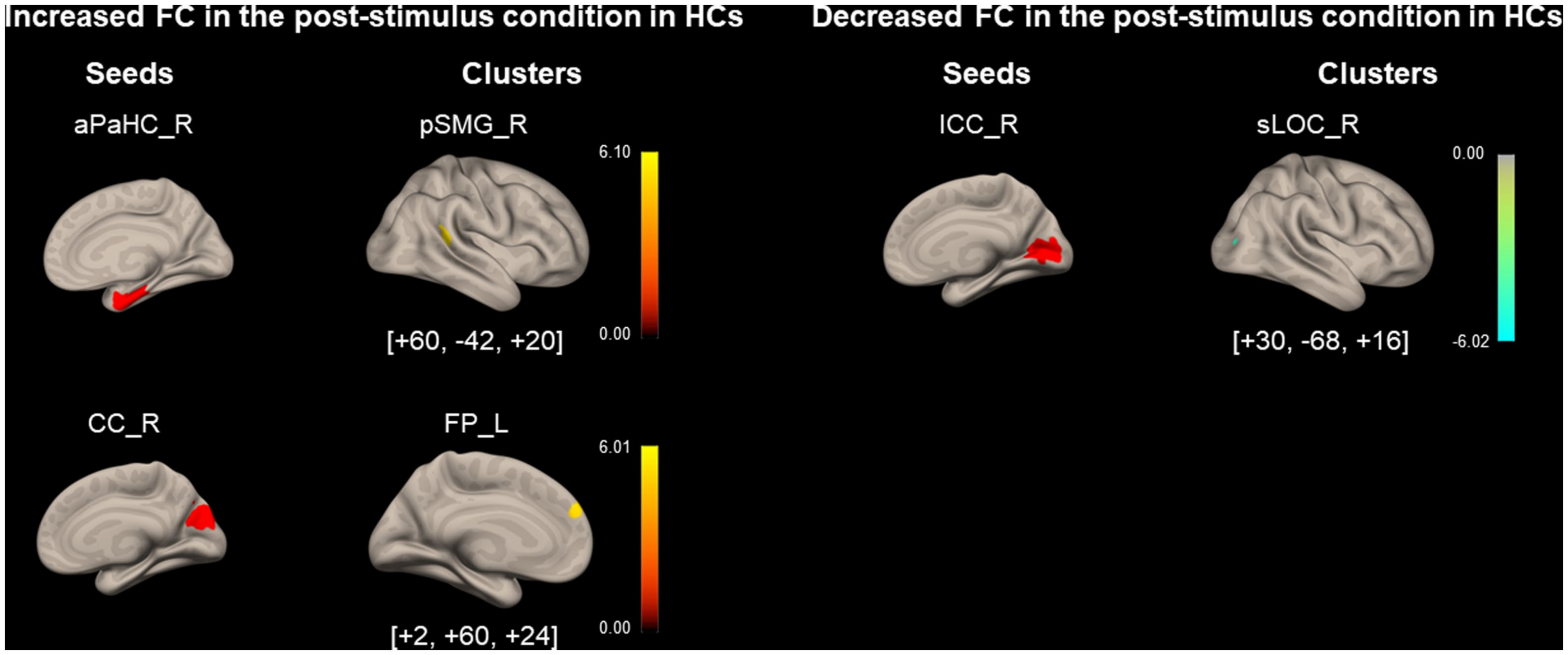
Significantly modified functional connectivity under the post-stimulus condition in the healthy control group. Seed regions are shown in red, increased functional connectivity (FC) is indicated by yellow bars, and decreased FC is indicated by green bars. The color bar represents *T* scores. The [*x*, *y*, *z*] values indicate the Montreal Neurological Institute (MNI) coordinates. aPaHC, anterior parahippocampal gyrus; CC, calcarine cortex; FP, frontal pole; HC, healthy control; ICC, intracalcarine cortex;pSMG, posterior supramarginal gyrus; sLOC, superior division of lateral occipital cortex.

As shown in Figure 5 and Table 5, FC between the right aPaHC and right pSMG, which was significantly lower in the HC group than in the PPPD group before the stimulus (Figure 3; Table 3), significantly increased in the post-stimulus condition than those in the pre-stimulus condition in the HC group. FC between the right CC and left FP significantly increased, whereas FC between the right ICC and right superior division of lateral occipital cortex (sLOC) was significantly decreased in the post-stimulus condition than that in the pre-stimulus condition in the HC group.

**Table 5:** Significantly modified functional connectivity (FC) in the post-stimulus condition in the healthy controls (HCs).

Seed region	Cluster coordinates (x, y, z)	Cluster size	Cluster regions	Cluster *value of p* (FWE)
*Increased FC*				
Parahippocampal Gyrus, anterior division_R	+60–42 + 20	113	Supramarginal Gyrus, posterior division_R	<0.01
Cuneal Cortex_R	+2 + 60 + 24	68	Frontal Pole_L	0.048
*Decreased FC*				
Intracalcarine Cortex _R	+30–68 + 16	134	Lateral Occipital Cortex, superior division_R	<0.01

FC, functional connectivity; HCs, healthy controls; FWE, family-wise error; L, left; R, right.

### 3.4. Differences between pre- and post-stimulus FCs in PPPD relative to HCs

**Figure 6:**
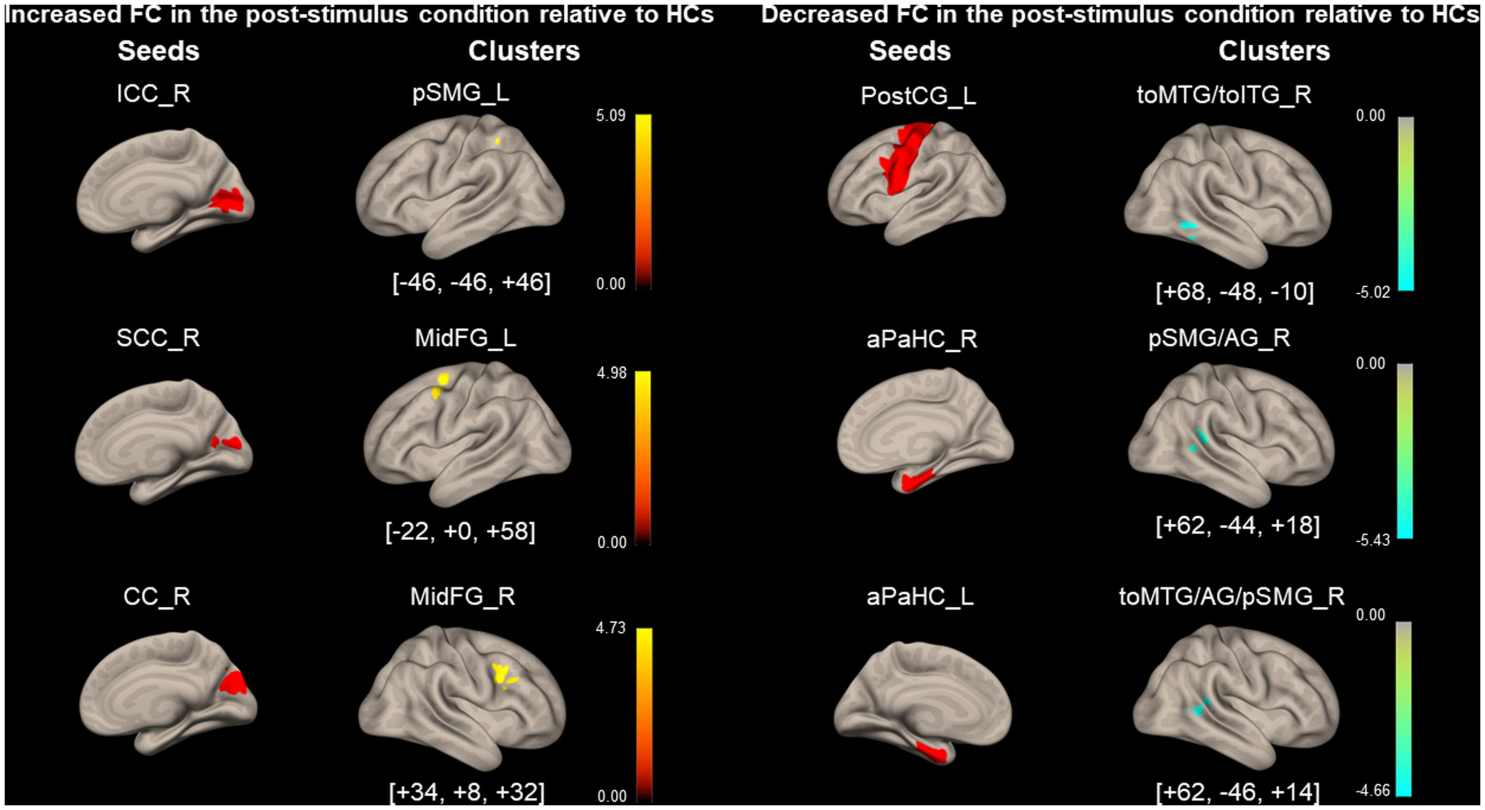
Significantly modified functional connectivity under the post-stimulus condition in patients with persistent postural-perceptual dizziness relative to that of healthy control. Seed regions are shown in red, increased functional connectivity (FC) is indicated by yellow bars, and decreased FC is indicated by green bars. The color bar represents *F* scores. The [*x*, *y*, *z*] values indicate the Montreal Neurological Institute (MNI) coordinates. aPaHC, anterior parahippocampal gyrus; CC, calcarine cortex; ICC, intracalcarine cortex; MidFG, middle frontal gyrus; PostCG, post-central gyrus; PPPD, persistent postural-perceptual dizziness; pSMG/AG, posterior supramarginal gyrus/angular gyrus; SCC, supracalcarine cortex; toMTG/toITG, temporooccipital middle/inferior temporal gyrus.

Figure 6 and Table 6 show the FCs modulated by visual stimulation in the PPPD group relative to the HC group. FC between the right ICC and the left pSMG, the right SCC and the left middle frontal gyrus (MidFG), and the right CC and the right MidFG significantly increased in the post-stimulus condition in the PPPD group relative to the HC group. It should be noted that this was an increase relative to that in the HC group. Since these three FCs neither occurred in the list of increased FC after visual stimulus in the PPPD group (Figure 4; Table 4) nor in that of decreased FC after visual stimulus in the HC group (Figure 5; Table 5), increase in these FCs could be derived from the combination of increase and decrease in FC of the PPPD and HC groups, respectively (See Methods and Figure 2). FC between the left PostCG and the right toMTG/toITG, the right aPaHC and the right pSMG/AG, and the left aPaHC and the right toMTG/AG/pSMG significantly decreased in the PPPD group compared with that in the HC group (Figure 6; Table 6). Among these, FC between the right aPaHC and the right pSMG/AG was higher in the PPPD group than that in the HC group in the pre-stimulus condition (Figure 3; Table 3). There were no significant changes in this FC between pre- and post-stimulus conditions in the PPPD group (Figure 4; Table 4), while this FC significantly increased in the post-stimulus than in the pre-stimulus condition in the HC group (Figure 5; Table 5). In summary, the relative decrease in FC observed in the post-stimulus condition of PPPD may imply that this FC increased after visual stimulation in the HC group; however, it could no longer occur in the PPPD group, perhaps this FC had already been fully facilitated even in the pre-stimulus condition.

**Table 6:** Significantly modified functional connectivity (FC) in the post-stimulus condition in persistent postural-perceptual dizziness (PPPD) relative to healthy controls (HCs).

Seed region	Cluster coordinates (x, y, z)	Cluster size	Cluster regions	Cluster *value of p* (FWE)
*Increased FC*				
Intracalcarine Cortex_R	− 46 -46 + 46	96	Supramarginal Gyrus, posterior division_L	0.047
Supracalcaraine Cortex _R	− 22 + 0 + 58	168	Middle Frontal Gyrus_L	<0.01
Cuneal Cortex_R	+ 34 + 8 + 32	105	Middle Frontal Gyrus_R	0.026
*Decreased FC*				
Postcentral Gyrus_L	+ 68– 48 – 10	118	Middle/Inferior Temporal Gyrus, temporooccipital part_R	0.017
Parahippocampal Gyrus, anterior division_R	+ 62–44 + 18	167	Supramarginal Gyrus, posterior division_R/Angular Gyrus_R	<0.01
Parahippocampal Gyrus, anterior division_L	+ 62– 46 + 14	111	Middle Temporal Gyrus, temporooccipital part_R/Angular Gyrus_R/Supramarginal Gyrus, posterior division_R	0.022

FC, functional connectivity; L, left; PPPD, persistent postural-perceptual dizziness; R, right; HCs, healthy control; FWE, family-wise error.

### 3.5. Brain activity during visual stimulation in PPPD: task-based fMRI analysis

No areas were significantly activated/inhibited during all five visual stimulations in the PPPD group compared with those in the HC group (data not shown).

## 4. Discussion

### 4.1. Demographic and clinical characteristics of PPPD

As shown in Table 1, the PPPD group had higher total scores for HADS and neuroticism scores for TIPI-J than the HC group. These psychiatric trends of patients with PPPD, i.e., anxiety/depression and neuroticism are consistent with those in previous reports (8, 31).

The PPPD group had broader elliptical balance areas on posturography than the HC group under the eyes closed, eyes open on foam rubber, and eyes closed on foam rubber conditions. These posturography results are consistent with previous outcomes (13, 14), showing no significant differences under the eyes open condition compared with that in the HC group, but significantly poorer performance under challenging conditions such as the eyes closed or standing on foam rubber conditions. Therefore, postural stability, which is barely maintained under the eyes open condition, would be easily disrupted by mild stimulation in patients with PPPD.

Regarding the vestibular function, the comprehensive findings of the examination revealed no cases of obvious unilateral or bilateral vestibular dysfunction, consistent with previous reports (32, 33) that described a deficit of specific laboratory findings.

SSQ (total score) was significantly higher in the PPPD group, indicating that patients with PPPD were more likely to be affected by motion sickness symptoms by visual stimuli (Table 1). Vestibular symptoms in patients with PPPD were considered to be exacerbated by visual stimuli and symptoms such as nausea and disorientation also occurred in conjunction with the exacerbation.

In summary, although the number of participants in this study was relatively small; the demographic and clinical features of patients with PPPD, e.g., anxious/depressive, neurotic, unstable posture, almost normal canal function, and susceptibility to visual stimuli were consistent with those of previous studies.

### 4.2. Comparison of FCs between PPPD and HCs at rest

Regarding the visuo- and vestibulo-spatial cognitive processes, the right hemisphere may be the dominant hemisphere (34, 35). As shown in Figure 3 and Table 3, significant differences in FC with seeds of PIVC, aPaHC, and HC of the dominant hemisphere were observed between the HC and PPPD groups: FC between right PIVC and bilateral LGs, that between the right aPaHC and right inferior parietal lobule (pSMG/AG), and that between the right HC and left FP.

Significantly lower FC was found in the PPPD group than in the HC group between the right PIVC, vestibular cortex, and bilateral LGs, the visual areas (Figure 3; Table 3). This is consistent with previous reports where decreased FC was found between the vestibular cortex, represented by the posterior perisylvian regions, and visual areas such as the extrastriate areas when evaluating FC in patients with PPPD or predecessors of PPPD relative to HCs (36, 37). Moreover, in our study, a higher FC was observed between the PostCG of the left dominant side (38), a somatosensory cortex, and toMTG/toITG of the right dominant side (39), upstream of the visual pathway (Figure 3; Table 3). Li et al. (40) also found a similar increase in FC between the post-central gyrus and the occipital pole visual network in PPPD. These findings suggest that vestibular inputs are not fully utilized in the vestibulo-visuo-somatosensory network, and the somatosensory and visual inputs would compensate for the vestibular inputs, leading to visually and somatosensory-dependent maintenance of spatial orientation in PPPD.

The PPPD group showed significantly higher FC between the right aPaHC and the right inferior parietal lobule (pSMG/AG) than the HC group (Figure 3; Table 3). The inferior parietal lobule including pSMG/AG is a spatial cognitive area that aggregates and integrates multiple types of sensory information and regulates the spatial interrelationship between the body and the external environment (41, 42), while the aPaHC is involved in visuospatial processing (43). Therefore, visual inputs are more likely facilitated in patients with PPPD than in HCs to maintain spatial cognition.

FC between the right HC and the left FP was significantly higher in the PPPD group than in the HC group (Figure 3; Table 3). Since HC and FP are the central areas for spatial cognition and mood control, respectively, the facilitation of this FC would account for changes in mood, e.g., anxiety, induced by tasks that require the spatial cognitive processes in patients with PPPD.

FCs between the right PostCG and the left FP and that between the left PIVC and the left FP/PCG were significantly lower in the PPPD group than in the HC group under the pre-stimulus condition (Figure 3; Table 3). Although the finding was significant, the role of differences in FCs from these non-dominant seed regions (right PostCG and left PIVC) should be treated with caution.

### 4.3. Brain activity during visual stimulation in PPPD

Task-based fMRI demonstrated that there were no brain regions that showed significantly different activities between the HC and PPPD groups during all five visual stimulations (data not shown), suggesting that there was no difference in the visual processing in PPPD and HCs. Consistent with our results, Riccelli et al. (44) found no significant difference in brain activity between the HC and PPPD groups assessed by fMRI when presented with virtual-reality rollercoaster stimuli in the motion vs. static conditions, whereas when vertical vs. horizontal motion conditions were compared, they found greater activation in the third short insular gyrus and adjacent Rolandic operculum in the HC group than that in the PPPD group. Although some studies previously demonstrated visually activated/inhibited areas in PPPD (45, 46), our results failed to reveal significant areas that were affected during visual stimuli relative to that in HCs. This could be attributed to the fact that although five types of visual stimuli were used in this study, the degree of symptom exacerbation by each stimulus would vary from patient to patient, and the diversity of symptoms within the disease group, which is also observed in real clinical practice, may have prevented the demonstration of the significant areas.

### 4.4. FCs in HCs and PPPD after visual stimulations

FC between the right aPaHC and right pSMG, which was higher in the PPPD group than in the HC group under the pre-stimulus condition (Figure 3; Table 3), increased under the post-stimulus condition in the HC group (Figure 5; Table 5). In contrast, this FC could no longer be increased under the post-stimulus condition in the PPPD group (Figure 4; Table 4); rather, it decreased relative to that in the HC group (Figure 6; Table 6), perhaps because this FC had already been elevated under the pre-stimulus condition in the PPPD group. An increase in this FC under the post-stimulus condition in the HC group suggests that visuospatial pathways were facilitated in the spatial cognitive processes even in HCs after visual stimulation. Given that the vestibular symptoms were never induced in the HC group during/after visual stimulation, enhancement of only this FC was not sufficient to account for visually dependent spatial orientation nor visual exacerbation of symptoms. Additional facilitation of FC between somatosensory (PostCG) and visual (toMTG/toITG; Figure 3; Table 3) areas to that between aPaHC and pSMG/AG might be responsible for the visual exacerbation in PPPD.

FCs from several seed regions of visual areas of the dominant side (47), e.g., right ICC/SCC/CC, increased under the post-stimulus condition in the PPPD group compared with that in the HC group. FC between the right ICC and the left pSMG increased under the post-stimulus condition compared with that under the pre-stimulus condition in the PPPD group relative to the HC group (Figure 6; Table 6). Since ICC and pSMG were the centers for visual processing and spatial cognition, respectively, it is suggested that visuospatial pathways were facilitated in PPPD after visual stimulation. FC between the right SCC/CC and the left/right MidFG, the prefrontal responsible area for emotion and mood disorders (48, 49), also increased under the post-stimulus condition in the PPPD group relative to the HC group (Figure 6; Table 6). Popp et al. (45) and Passamonti et al. (50) also reported an increase in FC by visual stimulations between the visual and prefrontal areas in PPPD. All these results would account for the prolonged symptoms after a visual exacerbation and anxious status in patients with PPPD.

FC between the left PostCG and the right toMTG/toITG, which was higher in the PPPD group than in the HC group under the pre-stimulus condition (Figure 3; Table 3), significantly decreased under the post-stimulus condition in the PPPD group relative to the HC group (Figure 6; Table 6). Since changes of this FC were not observed in the HC group (Figure 5; Table 5), it is suggested that visual stimulation would weaken the somatosensory (postCG) to visual (toMTG/toITG) circuit of spatial orientation, which was heightened even at rest in patients with PPPD. In clinical settings, this may imply that the vestibular rehabilitation that promotes habituation to visual stimuli would effectively affect this point in the treatment of PPPD.

Significance of changes observed under the post-stimulus condition in the PPPD group, e.g., an increase in FC between the vestibular (PIVC) and visual (LG) areas and that between the visual (SCC) and spatial cognitive (pSMG) areas and a decrease in FC among the visual areas (CC and pMTG; Figure 4; Table 4), disappeared when analyzed relative to HCs (Figure 6; Table 6). Therefore, these data must be interpreted carefully. FC between the left aPaHC and the right toMTG/AG/pSMG decreased under the post-stimulus condition in the PPPD group relative to the HC group (Figure 6; Table 6). FC between the right PostCG and the right pSMG also decreased under the post-stimulus condition compared with that under the pre-stimulus condition in the PPPD group. Although the results were significant, the role of differences in FCs from these non-dominant seed regions (left aPaHC and right PostCG) should be treated with caution.

### 4.5. Neural mechanisms underlying PPPD

**Figure 7:**
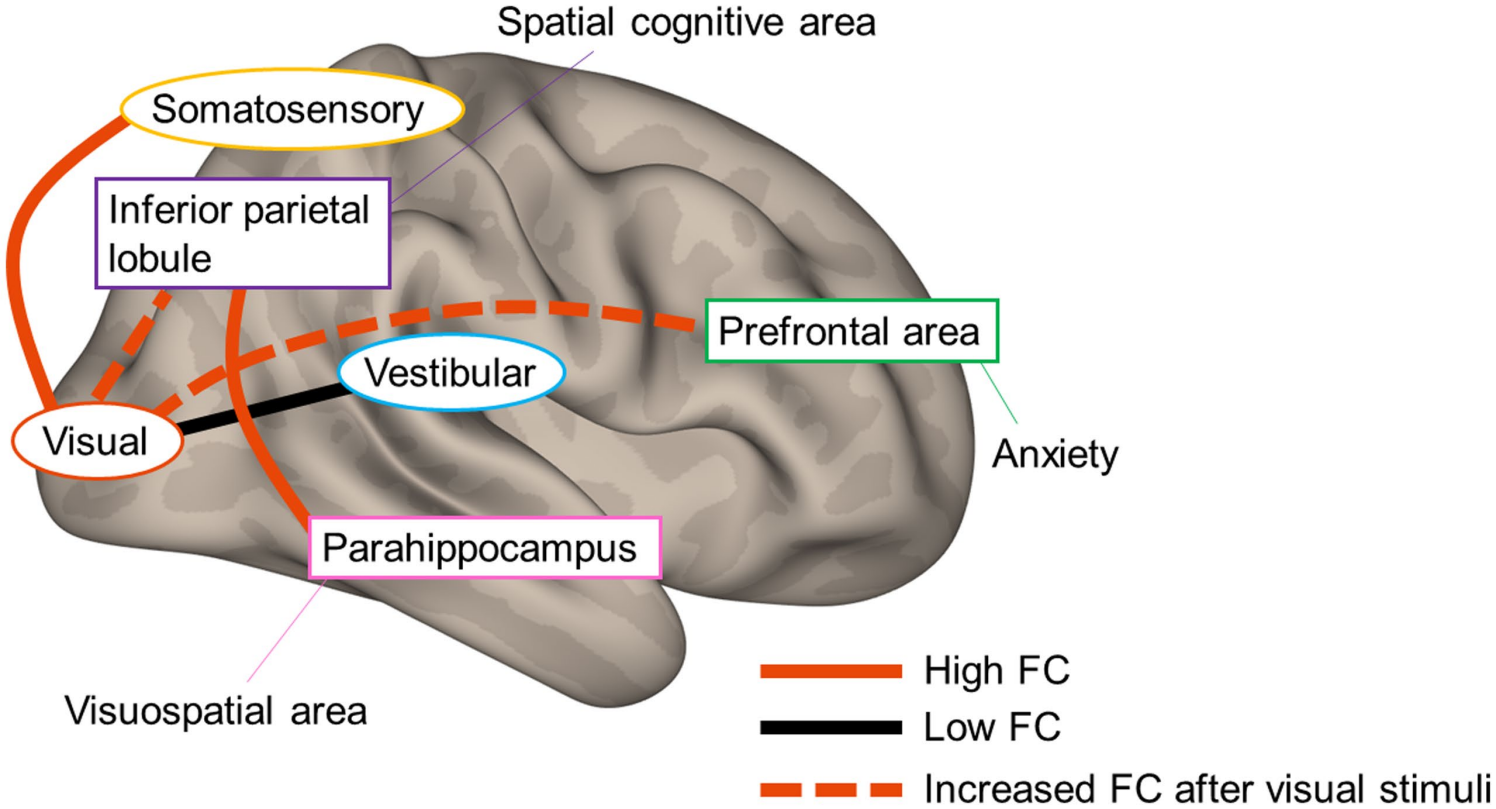
Neural mechanisms underlying persistent postural–perceptual dizziness. The solid red line and black line indicate high functional connectivity (FC) and low FC, respectively. The red dotted line indicates increased FC after visual stimuli.

Figure 7 summarizes the current results and possible neural mechanisms underlying PPPD. At rest, while FC between vestibular and visual cortices is low, that between somatosensory and visual cortices is high, suggesting that vestibular inputs are not fully utilized in the vestibulo-visuo-somatosensory network. A heightened FC between parahippocampal visuospatial and spatial cognitive areas of the inferior parietal lobe in combination with visually and somatosensory-dependent spatial orientation strategy would be involved in the visual exacerbation in PPPD. An increase in FC from visual areas to spatial cognitive and prefrontal areas after visual stimuli may account for the prolonged symptoms after a visual exacerbation and anxious status in PPPD. Overall, the study presents the underlying neural mechanisms involved in PPPD and will promote better management of the patients.

### 4.6. Limitations

There are several limitations in this study. First, the psychological factors were not controlled due to the small sample size. Second, patients on antidepressants were included (51–53). Third, the possibility that brain regions other than the seed region used in this analysis may be implicated in the pathogenesis of PPPD cannot be denied. Lastly, it was difficult to determine whether the FC changes observed in this study were a cause or a consequence. To better elucidate the pathogenesis of PPPD, it is necessary to interpret not only the results of fMRI studies but also combine them with the results of clinical tests, such as the sensory organization test, subjective visual vertical test, eye-tracking test, or spatial cognition test.

## 5. Conclusion

In PPPD, vestibular inputs may not be fully utilized in the vestibulo-visuo-somatosensory network. The FC between visuospatial and spatial cognitive areas was increased even in HCs after visual stimuli. Hence, the elevated status of this FC in combination with the high FC between the somatosensory and visual areas would be involved in the visual exacerbation in PPPD. An increase in FC from the visual areas to spatial cognitive and prefrontal areas after visual stimuli may account for the prolonged symptoms after a visual exacerbation and anxious status in PPPD.

## Data availability statement

The raw data supporting the conclusions of this article will be made available by the authors, without undue reservation.

## Ethics statement

The studies involving human participants were reviewed and approved by the institutional review board of Niigata University Medical and Dental Hospital. The patients/participants provided their written informed consent to participate in this study.

## Author contributions

CY: conceptualization, methodology, formal analysis, investigation, data curation, writing—original draft, writing—review and editing, project administration, and funding acquisition. YM, TY, SO, SI, KT: resources, writing—review and editing. MW: software, investigation, and resources. KI: methodology, formal analysis, investigation, writing—original draft, and writing—review and editing. YS: methodology, formal analysis, investigation, writing—review and editing, and project administration. HI: writing—review and editing, and supervision. AH: conceptualization, resources, writing—original draft, writing—review and editing, supervision, and funding acquisition. All authors contributed to the article and approved the submitted version.

## Funding

This work was partly supported by Grants-in-Aid from the Ministry of Education, Culture, Sports, Science, and Technology of Japan [19K18799] (for CY) and [21H03084] (for AH).

## Conflict of interest

The authors declare that the research was conducted in the absence of any commercial or financial relationships that could be construed as a potential conflict of interest.

## Publisher’s note

All claims expressed in this article are solely those of the authors and do not necessarily represent those of their affiliated organizations, or those of the publisher, the editors and the reviewers. Any product that may be evaluated in this article, or claim that may be made by its manufacturer, is not guaranteed or endorsed by the publisher.

## Abbreviations

PPPD: persistent postural-perceptual dizziness
FC: functional connectivity
HCs: healthy controls
rs-fMRI: resting-state fMRI
DHI: dizziness handicap inventory
HADS: hospital anxiety and depression scale
TIPI-J: the Japanese version of the Ten Item Personality Inventory
VAS: visual analog scale
SSQ: simulator sickness questionnaire
RCT: rotatory chair test
vHIT: video head impulse test
cVEMP: cervical vestibular evoked myogenic potentials
oVEMP: ocular vestibular-evoked myogenic potential
SVV: subjective visual vertical
CP: canal paresis
VOR: vestibulo-ocular reflex
CUS: catch-up saccades
IAARs: interaural asymmetry ratios
3D-FSPGR: three-dimension spoiled gradient recalled echo
TR: repetition time
FOV: field of view
TE: echo time
MNI: Montreal neurological institute
FWHM: fullwidth at half-maximum
PIVC: parieto-insular vestibular cortex
PIC: posterior insular cortex
ICC: intracalcarine cortex
SCC: supracalcarine cortex
LG: lingual gyrus
CC: cuneal cortex
PostCG: post-central gyrus
aPaHC and pPaHC: anterior/posterior parahippocampal gyrus
HC: hippocampus
FWE: family-wise error
IQR: interquartile ranges
AUVP: acute unilateral vestibulopathy
BPPV: benign paroxysmal positional vertigo
toMTG/toITG: temporooccipital part of middle/inferior temporal gyrus
pSMG/AG: posterior division of supramarginal gyrus/angular gyrus
FP: frontal pole
PCG: paracingulate gyrus
SFG: superior frontal gyrus
sLOC: superior division of lateral occipital cortex
MidFG: middle frontal gyrus
pSMG: posterior supramarginal gyrus
