# Supplementary material for: Changes in functional connectivity among vestibulo-visuo-somatosensory and spatial cognitive cortical areas in persistent postural-perceptual dizziness: resting-state fMRI studies before and after visual stimulation
Source: Front Neurol. 2023 Jul 24;14:1215004. doi: 10.3389/fneur.2023.1215004 (PMC10406134; doi:10.3389/fneur.2023.1215004)
Supplement: Supplementary file 1 [file Table_1.docx]

Supplementary Material

Changes in functional connectivity among vestibulo-visuo-somatosensory and spatial cognitive cortical areas in persistent postural-perceptual dizziness: Resting-state fMRI studies before and after visual stimulation

**Chihiro Yagi^1*^, Yuka Morita^1^, Tatsuya Yamagishi^1^, Shinsuke Ohshima^1^, Shuji Izumi^1^, Kuniyuki Takahashi^2^, Masaki Watanabe^3^, Kosuke Itoh^3^, Yuji Suzuki^3^, Hironaka Igarashi^3^, Arata Horii^1^**

*** Correspondence:** Chihiro Yagi, Email: [c-yagi@med.niigata-u.ac.jp](mailto:c-yagi@med.niigata-u.ac.jp)

**Supplemental Table 1. Clinical characteristics of patients with persistent postural-perceptual dizziness (PPPD)**

| Patient No. | P01 | P02 | P03 | P04 | P05 | P06 | P07 | P08 | P09 | P10 | P11 |
| --- | --- | --- | --- | --- | --- | --- | --- | --- | --- | --- | --- |
| **CP**  **(%)** | -22.4 | 26.1 | 15.5 | 11.4 | 6.5 | 29.6 | 10.8 | -0.8 | -25.7 | 18.4 | 19.8 |
| **Max. SPV**  **Rt. 26°C (°/s)** | 5.6 | 24.5 | 19.1 | 26.3 | 23.4 | 32.0 | 17.0 | 20.5 | 24.0 | 44.7 | 20.1 |
| **Max. SPV**  **Lt. 26°C (°/s)** | 14.6 | 13.8 | 12.1 | 16.0 | 18.0 | 18.7 | 10.3 | 37.7 | 32.4 | 31.3 | 11.9 |
| **Max. SPV**  **Rt. 45°C (°/s)** | 8.4 | 13.0 | 8.4 | 14.3 | 15.0 | 23.6 | 7.3 | 52.2 | 19.5 | 22.0 | 13.6 |
| **Max. SPV**  **Lt. 45°C (°/s)** | 7.5 | 8.1 | 8.0 | 16.3 | 15.7 | 11.5 | 9.3 | 36.3 | 41.2 | 14.7 | 10.6 |
| **RCT**  **Rt. VOR** | 0.85 | 0.56 | 0.36 | 0.76 | 0.32 | 0.58 | 0.50 |  | 1.0 | 0.60 | 0.37 |
| **RCT**  **Lt. VOR** | 0.75 | 0.59 | 0.45 | 0.6 | 0.32 | 0.74 | 0.45 |  | 1.0 | 0.65 | 0.62 |
| **vHIT-RL/**  **CUS (+ or -)** | 0.80 / (-) | 0.94 / (-) |  | 1.06 / (-) |  | 1.17 / (-) | 1.01 / (-) | 1.03 / (-) | 0.87 / (-) | 1.14 / (-) |  |
| **vHIT-LL/**  **CUS (+ or -)** | 0.88 / (-) | 1.06 / (-) |  | 0.98 / (-) |  | 1.04 / (-) | 1.09 / (-) | 0.96 / (-) | 1.10 / (-) | 1.15 / (-) |  |
| **vHIT-RA/**  **CUS (+ or -)** | 0.90 / (-) | 1.50 / (-) |  | 1.24 / (-) |  | -^*^ | 1.05 / (-) | 1.00 / (-) | 1.10 / (-) | 1.21 / (-) |  |
| **vHIT-LA/**  **CUS (+ or -)** | 1.01 / (-) | 1.95 / (-) |  | 1.45 / (-) |  | -^*^ | 1.23 / (-) | 1.26 / (-) | 1.41 / (-) | 1.87 / (-) |  |
| **vHIT-RP/**  **CUS (+ or -)** | 0.99 / (-) | 1.37 / (-) |  | 1.48 / (-) |  | -^*^ | 1.33 / (-) | 1.20 / (-) | 1.29 / (-) | 1.64 / (-) |  |
| **vHIT-LP/**  **CUS (+ or -)** | 0.64 / (-) | 1.28 / (-) |  | 1.21 / (-) |  | -^*^ | 0.96 / (-) | 0.82 /(+) | 0.97 / (-) | 1.09 / (-) |  |
| **cVEMP**  **IAAR** | -14.0 | -3.2 | -31.6 | -1.7 | 15.0 | -23.1 | 9.3 |  | Lt. Absent | 22.6 | -24.4 |
| **oVEMP**  **IAAR** | 38.9 | 1.4 | 10.9 | -22.4 | Absent | Absent | Absent |  | 2.7 | -20.2 | Absent |
| **SVV**  **(degree)** | -2.3 | 0.5 | -0.3 | -1.5 | -0.5 | -1.5 | 0.0 | 0.3 | 2.2 | -0.3 | 0.3 |

CP, canal paresis; CUS, catch-up saccades; cVEMP, cervical vestibular-evoked myogenic potential; IAAR, interaural asymmetry ratio; LA, left anterior canal; LL, left lateral canals; LP, left posterior canal; Lt., left; Max. SPV, maximum slow phase velocity; oVEMP, ocular VEMP; PPPD, persistent postural-perceptual dizziness; RA, right anterior canal; RCT, rotatory chair test; RL, right lateral canal; RP, right posterior canal; Rt., right; SVV, subjective visual vertical; vHIT, video head impulse test; VOR, vestibulo-ocular reflex

Negative values in CP, IAAR, and SVV indicate right-sided hypofunction.

^*^ indicates that the waveform could not be obtained due to eyelid narrowing.
